# Costs incurred by patients with drug-susceptible pulmonary tuberculosis in semi-urban and rural settings of Western India

**DOI:** 10.1186/s40249-020-00760-w

**Published:** 2020-10-19

**Authors:** Mihir P. Rupani, Adithya Cattamanchi, Priya B. Shete, William M. Vollmer, Sanjib Basu, Jigna D. Dave

**Affiliations:** 1 Department of Community Medicine, Government Medical College Bhavnagar (Maharaja Krishnakumarsinhji Bhavnagar University), Near ST Bus Stand, Jail Road, Bhavnagar, Gujarat 364001 India; 2 Division of Pulmonary and Critical Care Medicine and Center for Tuberculosis, University of California San Francisco (UCSF), California, USA; 3 Division of Biostatistics, Kaiser Permanente Center for Health Research, Portland, USA; 4 Division of Epidemiology and Biostatistics, School of Public Health, University of Illinois at Chicago, Chicago, USA; 5 Department of Respiratory Medicine, Government Medical College Bhavnagar (Maharaja Krishnakumarsinhji Bhavnagar University), Bhavnagar, Gujarat India

**Keywords:** Tuberculosis cost tool, Catastrophic cost, Healthcare cost, Treatment outcome, National tuberculosis program, India, Coping, Cash transfer, National tuberculosis elimination program, American thoracic society-methods in epidemiologic, clinical, and operations research

## Abstract

**Background:**

India reports the highest number of tuberculosis (TB) cases worldwide. Poverty has a dual impact as it increases the risk of TB and exposes the poor to economic hardship when they develop TB. Our objective was to estimate the costs incurred by patients with drug-susceptible TB in Bhavnagar (western India) using an adapted World Health Organization costing tool.

**Methods:**

We conducted a descriptive cross-sectional study of adults, notified in the public sector and being treated for drug-susceptible pulmonary TB during January–June 2019, in six urban and three rural blocks of Bhavnagar region, Gujarat state, India. The direct and indirect TB-related costs, as well as patients’ coping strategies, were assessed for the overall care of TB till treatment completion. Catastrophic costs were defined as total costs > 20% of annual household income (excluding any amount received from cash transfer programs or borrowed). Median and interquartile range (IQR) was used to summarize patient costs. The median costs between any two groups were compared using the median test. The association between any two categorical variables was tested by the Pearson chi-squared test. All costs were described in US dollars (USD). During the study period, on average, one USD equalled 70 Indian Rupees.

**Results:**

Of 458 patients included, 70% were male, 62% had no formal education, 71% lived in urban areas, and 96% completed TB treatment. The median (IQR) total costs were USD 8 (5–28), direct medical costs were USD 0 (0–0), direct non-medical costs were USD 3 (2–4) and indirect costs were USD 6 (3–13). Among direct non-medical costs, travel cost (median = USD 3, IQR: 2–4) to attend health facilities were the most prominent, whereas the indirect costs were mainly contributed by the patient’s loss of wages (median = USD 3, IQR: 0–6). Four percent of patients faced catastrophic costs, 11% borrowed money to cover costs and 7% lost their employment; the median working days lost to TB was 30 (IQR: 15–45). A majority (88%) of patients received a median USD 43 (IQR: 41–43) as part of a cash transfer program for TB patients.

**Conclusions:**

Treatment completion was high and the costs incurred by TB patients were low in this setting. However, negative financial consequences occur even in low-cost settings. The role of universal cash transfer programs in such settings requires further study.

## Background

India is the country with the largest share of the global burden of tuberculosis (TB) cases (27%), with the burden being highest among the poor [1]. Poverty has a dual impact as poor patients are more at risk of TB and may face greater economic hardship when they develop TB [1]. The World Health Organization (WHO) has suggested no TB patients should face catastrophic costs, defined as costs exceeding 20% of the annual household income, and have included it as a key indicator for TB control [2, 3]. National surveys of costs faced by TB patients are increasingly being done using WHO’s validated costing tool [2]. Studies from Mongolia, Fiji, the Philippines, Vietnam, Uganda, Ghana, Kenya, and Nigeria found 27–70% of drug-susceptible TB patients incur catastrophic costs [1, 4–7]. A study in Peru has established the association of catastrophic health costs with failure to complete treatment [8].

Studies in India reported the percentage of catastrophic costs among drug-susceptible TB patients to be between 7 and 32% [9–12]. The average costs incurred by drug-susceptible TB patients treated in government health facilities are approximately USD 179, [9, 10] whereas costs incurred by TB patients accessing care in all types of health facilities range from USD 20 to 224 [13–17]. In response to call for additional socioeconomic support for TB patients, the National TB Program (NTP) in India rolled out a direct benefit transfer (DBT) scheme in April 2018 with a credit of USD 7 (Indian Rupees [INR] 500) every month to support the nutritional requirements of TB patients [18]. However, only a few studies have assessed the complete costs incurred through treatment completion by TB patients in India using a validated WHO tool [9, 11, 12].

The primary objective of the study was to estimate the costs incurred by drug-susceptible pulmonary TB patients in a semi-urban and rural setting in western India following the introduction of the DBT scheme. The secondary objective was to estimate the proportion of households facing catastrophic costs and to determine the association between catastrophic costs and failure to complete treatment.

## Methods

### Study setting

The study was conducted in all six semi-urban and three rural “blocks” (administrative zones) of the Bhavnagar region of Gujarat state in the western part of India. The semi-urban blocks were part of Bhavnagar city (population ~ 0.6 million), and the rural blocks were drawn from the surrounding, largely agricultural, countryside. Agriculture and daily wage labor are the primary occupations of residents in rural and urban areas of the district, respectively. The TB case notification rate in Bhavnagar district from the public and private sector was 1040 and 780 per million population, respectively, in the year 2019. The care for TB is offered free of cost in the public sector in a decentralized model through a network of public health facilities, the district hospital being the tertiary-level facility. Once initiated on treatment, the medicines and other necessary follow-up care is delivered to the patients from a nearby public health facility. Patients with drug-susceptible pulmonary tuberculosis are given a treatment of six months – two months of intensive phase (to rapidly eliminate the bacilli) followed by four months of continuation phase (to eradicate the dormant bacilli).

### Design and participants

We conducted a descriptive cross-sectional study of patients enrolled for the treatment of drug-susceptible TB. We included patients ≥ 18 years on treatment for drug-susceptible pulmonary tuberculosis, registered under the public sector in all six urban blocks of Bhavnagar city, and three of 11 randomly-selected rural blocks (Sihor, Palitana, and Mahuva) of Bhavnagar district. We excluded patients who were previously treated or taking treatment under a private health facility or put on treatment for < 14 days [2].

### Recruitment

The list (treatment registers) of patients put on treatment is maintained in physical form by TB Health Visitors at the District TB Centre of Bhavnagar. TB health visitors are contracted by NTP for record-keeping of TB patients in geographical areas assigned to them. For each of the nine selected urban and rural blocks, a trained field investigator reviewed the list of patients put on treatment to identify those who appeared to be eligible for the study, and then visited their homes (accompanied by the local TB health visitor) to confirm eligibility and collect data. Enrollment occurred between January 2019 and June 2019. Once patients from all nine blocks were enrolled, the process was repeated in selected urban blocks till June 2019 (end of the study period).

### Data collection

Following written informed consent, the field investigator administered a survey to collect basic demographic and clinical information (some of which was extracted from the TB treatment registers), and administered the adapted WHO costing tool [2]. The WHO costing tool included direct medical costs, direct non-medical costs, and indirect costs incurred by the patients for TB care. Apart from the cost questions, the tool also had questions eliciting the income, household ownership of selected assets, coping strategies employed by, and social consequences on the family.

We collected data on costs relative to the following time points based on patient recall: pre-diagnosis (the period from onset of symptoms until diagnosis), at the time of diagnosis, at the end of one-and-a-half month for adverse drug reaction or directly observed treatment (DOT) medicine collection visits, at the end of two months (first follow-up visit) and at the end of six months (treatment completion visit). The survey was administered at the homes of patients and covered all costs up until the time of enrollment in the study, that is, the patients were enrolled at variable time points of treatment. Depending on how far the patient was in the treatment phase, the field investigator later made up to three phone calls to collect data on the additional costs incurred until treatment completion.

The questionnaire was translated into the local language (Gujarati) by a language translator. Compensation of USD 0.7 (INR 50) was given to each participant for their time after completion of the interview. Patients were then followed-up passively to obtain treatment outcomes. The field investigator extracted data on treatment outcomes and DBT from the TB treatment registers and NIKSHAY (an online database for registered TB patients, https://www.nikshay.in/) respectively.

### Definitions

#### Cost data

All costs were summed over various time points. Direct medical costs were categorized as: costs incurred for hospital day charges, consultation charges, and costs for radiography, laboratory, other procedures, drugs, and prescribed nutrition. Direct non-medical costs included: costs for travel, food, and accommodation (including that of an accompanying member) to attend health facility, and the travel costs for DOT visits. Indirect costs included patient’s and accompanying member’s loss of wages, and a measure to assess economic impact calculated as monthly family income before TB minus monthly family income at the time of interview. The total costs incurred were a sum of direct medical, direct non-medical and indirect costs, and were said to be catastrophic if they exceeded 20% of their annual household income (excluding the DBT amount). Negative financial coping strategies (e.g., borrowing money or selling assets) and social consequences (e.g., social exclusion) were also assessed. During the study period, on average, USD 1 equalled INR 70.

#### Socioeconomic status

To stratify patients based on socioeconomic status, data on ownership of assets including the type of house, number of rooms, television, and others were used to create a standard of living (SLI) index. Depending on the ownership of different assets, a summary score (range: 1–23) adapted from the National Family Health Survey (NFHS), was calculated [19, 20]. SLI index score of 1–7 was considered as low SLI and 8–23 was considered as middle/high SLI.

#### Treatment outcome

Failure to complete treatment was defined according to NTP standards, including loss to follow-up (stopped treatment for at least one consecutive month), sputum smear positivity at the end of treatment, or death while on treatment [21].

### Data analysis

We collected data on all eligible patients during the study period of January–June 2019. The data were entered into a computerized form and analyzed in the statistical software EpiInfo 7.2.4.0 (Centre for Disease Control and Prevention, Atlanta, Georgia, USA). The total costs and its sub-components incurred by patients were described in median with inter-quartile range (IQR). The median costs between any two groups were compared using the median test. The association between catastrophic costs and failure to complete treatment was tested by the Pearson chi-squared test. We conducted sensitivity analyses using 15, 10, and 5% of one’s annual self-reported household income to define catastrophic costs.

## Results

### Study population

**Fig. 1:**
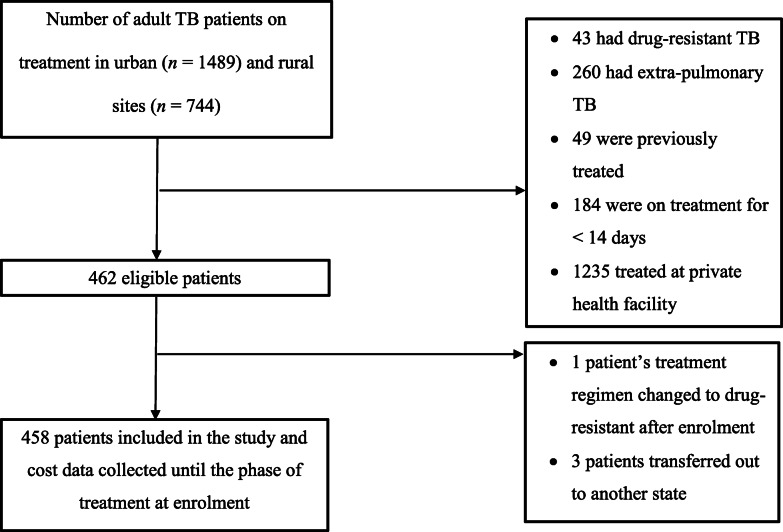
Selection process of patients on treatment for drug-susceptible pulmonary TB from six urban and three rural sites during January–June 2019 in Bhavnagar

Out of the 2233 total patients, 462 patients were eligible for the study (Fig. 1). Out of those eligible, four patients were removed from the final analysis. The response rate was 100% (all patients agreed to participate in the study).

### Characteristics of the study population

The median age of the patients was 35 (IQR: 23–50) years and the median number of family members was 5 (IQR: 4–6). Out of the 458 patients, 70% were male, 62% had no formal education, 72% were married, 71% lived in urban areas and 17% lived in a nuclear family (i.e., lived separately from parents of the primary wage earner) (Table 1). Eighty-eight percent of patients received DBT during the course of treatment (median = USD 43, IQR: 41–43). The median family income of the patients in USD was 129 (IQR: 100–186). Among the study participants, 141 (31%) had low SLI index, 204 (45%) were currently in paid work and 85 (19%) were sole earners in the family. Out of the 458 patients, the sputum smear was positive in 76% of patients, 13% had visited a private practitioner in their first visit and 14% were hospitalized due to TB in their first visit. The median number of days into treatment at the time of enrolment in the study was 69 (IQR: 29–116) days. The proportion of patients who failed to complete treatment was low at 4% (95% *CI*: 3–6%).

**Table 1:** Characteristics of patients on treatment for drug-susceptible pulmonary tuberculosis in Bhavnagar during January–June 2019 (*n* = 458)

Characteristic	Number (%) or median (IQR)
**Socio-demographic characteristics**	
Age, years	35 (23–50)
Male	319 (70)
Educational status	
No formal education	284 (62)
Primary (7th pass)	114 (25)
Secondary (10th pass) and above	60 (13)
Married	332 (72)
Scheduled caste/scheduled tribe	142 (31)
Extended family (vs nuclear family)	381 (83)
Urban residence	325 (71)
**Economic characteristics**	
DBT received for this episode of TB	405 (88)
Amount of DBT received in USD	43 (41–43)
Family income in USD	129 (100–186)
Below poverty line card	146 (32)
Standard of living (SLI) index	
Low (SLI score 1–7)	141 (31)
Middle/high (SLI score 8–23)	317 (69)
Employed in paid work before TB diagnosis	259 (57)
Currently in paid work	204 (45)
Sole earner in the family	85 (19)
**Clinical characteristics**	
Sputum acid-fast bacillus smear grade	
Negative	112 (25)
Scanty	70 (15)
1+	126 (27)
2+	50 (11)
3+	100 (22)
HIV positive	8 (2)
Diabetes	35 (8)
Current tobacco smoking	119 (26)
Current regular alcohol consumption	20 (4)
Intensive phase of TB treatment	195 (43)
First TB visit with a private provider	60 (13)
Hospitalized due to TB at the first visit	66 (14)
Number of days into TB treatment at the time of study enrolment	69 (29–116)

*IQR* Inter-quartile range; *DBT* Direct benefit transfer; *HIV* Human immunodeficiency virus; *TB* Tuberculosis. *USD 1* = INR 70

### Direct costs

The direct medical costs were minimal for the majority of the patients (median = 0, IQR: 0–0) (Table 2). For the 87% of patients who had their first visit at a government provider these costs were zero, while for those who had their first visit at a private practitioner median direct medical costs were USD 30 (IQR: 10–76). Median direct non-medical costs were USD 3 (IQR: 2–4) and differed significantly between patients who first visited a private vs a government provider (median USD 4 vs 3, *P* <  0.001). Among direct non-medical costs, travel cost (median = USD 3, IQR: 2–4) to attend health facilities were the most prominent.

**Table 2:** Median (IQR) costs in USD, by type of provider in first visit, incurred by patients with drug-susceptible pulmonary TB on treatment during January–June 2019, in Bhavnagar (*n* = 458)

Categories of costs	Total (***n*** = 458) Median (IQR)	Private provider (***n*** = 60) Median (IQR)	Government provider (***n*** = 398) Median (IQR)	***P***-value
Direct medical	0 (0–0)	30 (10–76)	0 (0–0)	< 0.001
Day charges	0 (0–0)	3 (0–7)	0 (0–0)	< 0.001
Consultation	0 (0–0)	0 (0–0)	0 (0–0)	< 0.001
Radiography	0 (0–0)	4 (0–7)	0 (0–0)	< 0.001
Laboratory	0 (0–0)	4 (0–7)	0 (0–0)	< 0.001
Procedure	0 (0–0)	0 (0–0)	0 (0–0)	0.274
Drug	0 (0–0)	14 (5–55)	0 (0–0)	< 0.001
Prescribed nutrition	0 (0–0)	0 (0–0)	0 (0–0)	0.003
Direct non-medical	3 (2–4)	4 (2–5)	3 (2–4)	< 0.001
Travel to attend health facility	3 (2–4)	4 (2–5)	3 (2–3)	0.002
Food purchased to attend health facility	0 (0–0)	0 (0–0)	0 (0–0)	0.854
Accommodation to attend health facility	0 (0–0)	0 (0–0)	0 (0–0)	0.274
DOT costs (travel)	0 (0–0)	0 (0–0)	0 (0–0)	0.935
Indirect	6 (3–13)	20 (3–76)	4 (3–10)	< 0.001
Loss of wages to attend health facility	3 (0–6)	8 (0–21)	3 (0–6)	0.029
Wage loss of accompanying member	0 (0–3)	0 (0–19)	0 (0–3)	0.84
Household income loss due to TB	0 (0–0)	0 (0–27)	0 (0–0)	0.003
Total costs	8 (5–28)	61 (35–156)	7 (5–14)	< 0.001

*IQR* Inter-quartile Range; *DOT* Directly Observed Treatment; *USD 1 =* INR 70

### Indirect costs

The median indirect costs among the patients were USD 6 (IQR: 3–13) and differed significantly among patients between private vs government providers (USD 20 vs 4, *P* < 0.001). The indirect costs were mainly contributed by the patient’s loss of wages (median = USD 3, IQR: 0–6).

### Total costs

The median total costs incurred by the patients was low (USD 8, IQR: 5–28), but differed significantly among patients who first visited a private vs a government provider (USD 61 vs USD 7, *P* < 0.001). We found no significant difference in total costs between low and middle/high-income participants (median = USD 8 vs USD 8, *P* = 0.98, see Supplementary Table 1, Additional file 1).

### Catastrophic costs

The percentage of patients who faced catastrophic costs was 4% (95% *CI*: 3–6%) and remained low in sensitivity analyses using lower thresholds of annual household income to define catastrophic costs (Table 3). Since most patients (96%) completed treatment, we were unable to meaningfully assess the association between catastrophic costs and failure to complete treatment (*P* = 0.80, data not shown). However, the odds of experiencing catastrophic costs were higher among patients visiting first a private vs a government provider (odds ratio: 4.2, 95% *CI*: 1.6–11.3, *P* = 0.002, see Supplementary Table 2, Additional file 2).

**Table 3:** Percentage of households facing catastrophic costs at different percentages of annual household income in Bhavnagar during January–June 2019 (*n* = 458)

Cut-off percentage of annual household income	Percentage of households facing catastrophic costs (95% ***CI***)
20	4 (3–6)
15	5 (4–8)
10	7 (5–10)
5	14 (11–17)

*CI* Confidence interval

### Coping strategies and social consequences

Among the 458 patients, 18% of patients had employed at least one negative financial coping strategy (Table 4). Eleven percent of households had to borrow a median of USD 71 (IQR: 29–143) to cover costs incurred after starting TB treatment. Also, one (0.25%) patient’s household stopped the tuition of their child to cover the cost of illness and two (0.5%) patient’s family members left their job to take care of them during their illness. Seven percent of patients lost their employment; the median of the working days lost to TB was 30 (IQR: 15–45). Also, the spouse of one (0.25%) patient gave divorce and family of one (0.25%) patient stopped talking with the patient due to TB.

**Table 4:** Coping strategies and social consequences by patients on treatment for drug-susceptible pulmonary tuberculosis in Bhavnagar during January–June 2019 (*n* = 458)

Type of impact	Number (%) or median (IQR)
**Coping strategy**	
Coping strategy of any kind	82 (18)
Borrowed money as loan	51 (11)
Amount borrowed in USD	71 (29–143)
Lost employment after TB diagnosis	33 (7)
Started employment to cover costs of TB	4 (1)
Family member left job	2 (0.5)
Working days lost to TB	30 (15–45)
Stopped tuition of children	1 (0.25)
**Social consequences**	
Spouse gave divorce due to TB	1 (0.25)
Family stopped talking due to TB	1 (0.25)

*IQR* Inter-quartile range; *TB* Tuberculosis. *USD 1 =* INR 70

## Discussion

Patients in this study had relatively low TB-related costs. This was particularly true for the 87% of patients who first visited the public sector as opposed to a private health care provider since direct medical charges for the former were zero. Among patients treated by private providers, the median total costs were USD 61 (IQR: 35–156). As a result of the lower overall costs in this population, the percentage who faced catastrophic costs was also low. Despite these low overall costs, 18% of families reported having to employ at least one negative financial coping strategy to manage their anticipated and actual costs.

The median costs reported in the present study were lower than those reported in most other studies from India [9, 10, 12, 13, 16, 17, 22, 23]. The discrepancy might be because the current study estimated the costs among drug-susceptible pulmonary TB patients, whereas the published literature is inclusive of cost estimates on drug-resistant and extra-pulmonary TB patients. The course of treatment for the latter group is longer and more complicated as compared with drug-susceptible TB patients. In addition to the difference in groups studied, most of the previous studies were conducted in a metropolitan city in contrast to the present study setting of a semi-urban and rural area. Finally, the current study was conducted among patients who were taking treatment at a government health facility, nullifying the post-diagnostic direct medical costs which might have been incurred at a private health facility.

When compared with other high-burden countries [1, 4–7], the present study reported a low percentage of households facing catastrophic costs. There is a possibility of underestimation of patient costs. Evidence suggests that the self-reported annual household incomes are lower than estimates based on assets, consumption, or expenditure [24]. However, most studies in other countries also used self-reported income and the percentage of households facing catastrophic costs found in the present study were comparable to a recent study conducted in New Delhi [9]. In contrast to the present study, a study in Peru found a significant association between catastrophic costs and adverse treatment outcomes among tuberculosis patients [8]. However, their research was conducted among drug-resistant TB patients who are more likely to incur higher costs and more likely to experience adverse treatment outcomes as compared with drug-susceptible TB patients [25, 26].

Around one-fifth of the patients in the current study employed a negative financial coping strategy to cover the costs of TB care. Negative financial coping strategies, also called dissavings, are directly associated with costs incurred by TB patients and may be used as a proxy indicator of the financial protection mechanisms being employed by governments [27]. Further program evaluation and implementation research are required to assess how cash transfer schemes like DBT can be better targeted to affect these outcomes [28, 29].

Several aspects of the study context likely affected these results. Study setting being a small town, patients faced less direct non-medical costs (costs for travel, food, accommodation, etc.) due to proximity to public health facilities. The setting where the present study was conducted has a wide network of government-run diagnostic and treatment facilities for tuberculosis. The public-private partnership model for TB control seems to be effective in Bhavnagar as the majority of patients had their first or second visit at a public health facility. The private practitioners seem to be aware of referring patients to public health facilities for reducing their economic hardships. Even though patients have to collect their medicines weekly, their medicine boxes are placed somewhere near to their homes (either a family member, neighbor, private practitioner, or health worker act as the DOT provider). This decentralized model of DOT helps to minimize direct non-medical costs incurred for such visits.

This was a single-center study in a semi-urban and rural setting with a well-functioning community-based DOT program that is achieving high levels of treatment success. Therefore the findings should only be generalized to similar settings in India. Even though this is one of the initial studies from India using the validated WHO tool [2] and adheres to the guidelines of reporting cross-sectional studies [30], it has some limitations. Some like recall bias and assessing costs after treatment completion are inherent to costing studies and were unavoidable, but, could have contributed to costs being underestimated.

## Conclusions

We conclude from the study that TB patients in our region incur minimal costs, perhaps due to the highly decentralized provision of diagnostic and treatment services through community-based DOT. These findings support the further expansion of community-based DOT models to reduce catastrophic costs in India and other countries. However, almost one in five participants undertook negative financial coping strategies to facilitate TB care, suggesting that negative financial consequences occur even in low-cost settings. Further research is needed to assess whether universal cash transfer programs achieve social protection targets in settings with well-functioning community-based DOT programs.

## Supplementary information

**Additional file 1.** Median costs in USD by standard of living index. Table showing median (with inter-quartile range) costs incurred by 458 patients with drug-susceptible pulmonary tuberculosis on treatment during January–June 2019.

**Additional file 2.** Association between type of provider at first visit and catastrophic costs. Table showing statistical association between type of provider at first visit and catastrophic costs incurred by 458 patients with drug-susceptible pulmonary tuberculosis on treatment during January–June 2019.

## Data Availability

The datasets generated and/or analysed during the current study are available in the Mendeley repository, https://data.mendeley.com/datasets/t3gb7f6xrx/1

## Abbreviations

*CI*: Confidence interval
DBT: Direct benefit transfer
DOT: Directly observed treatment
ICMR: Indian Council of Medical Research
INR: Indian Rupee
IQR: Inter-quartile range
NFHS: National Family Health Survey
NTP: National TB Program
SLI: Standard of Living index
TB: Tuberculosis
USD: United States Dollars
WHO: World Health Organization
