# Supplementary material for: Costs incurred by patients with drug-susceptible pulmonary tuberculosis in semi-urban and rural settings of Western India
Source: Infect Dis Poverty. 2020 Oct 19;9:144. doi: 10.1186/s40249-020-00760-w (PMC7574230; doi:10.1186/s40249-020-00760-w)
Supplement: Supplementary file 1 — Additional file 1. Median costs in USD by standard of living index. Table showing median (with inter-quartile range) costs incurred by 458 patients with drug-susceptible pulmonary tuberculosis on treatment during January–June 2019. [file 40249_2020_760_MOESM1_ESM.docx]

Supplementary Table 1: Median (IQR) costs in USD, by standard of living index, incurred by patients with drug-susceptible pulmonary tuberculosis on treatment during January–June 2019, in Bhavnagar (*n* = 458)

| **Costs** | **Total (*n* = 458)**  **Median (IQR)** | **Low SLI (*n* = 141)**  **Median (IQR)** | **Middle/high SLI (*n* = 317)**  **Median (IQR)** | ***P*-value** |
| --- | --- | --- | --- | --- |
| Direct medical | 0 (0–0) | 0 (0–0) | 0 (0–0) | 0.11 |
| Day charges | 0 (0–0) | 0 (0–0) | 0 (0–0) | 0.123 |
| Consultation | 0 (0–0) | 0 (0–0) | 0 (0–0) | 0.589 |
| Radiography | 0 (0–0) | 0 (0–0) | 0 (0–0) | **0.016** |
| Laboratory | 0 (0–0) | 0 (0–0) | 0 (0–0) | **0.037** |
| Procedure | 0 (0–0) | 0 (0–0) | 0 (0–0) | 0.677 |
| Drug | 0 (0–0) | 0 (0–0) | 0 (0–0) | 0.185 |
| Prescribed nutrition | 0 (0–0) | 0 (0–0) | 0 (0–0) | 0.426 |
| Indirect medical | 8 (5–13) | 8 (4–14) | 8 (5–13) | 0.988 |
| Loss of wages to attend health facility visits | 3 (0–6) | 3 (0–6) | 3 (0–7) | 0.482 |
| Travel to attend health facility visits | 3 (2–4) | 3 (2–4) | 3 (2–4) | 0.585 |
| Food purchased to attend health facility visits | 0 (0–0) | 0 (0–0) | 0 (0–0) | 0.469 |
| Accommodation to attend health facility visits | 0 (0–0) | 0 (0–0) | 0 (0–0) | 0.677 |
| Wage loss of accompanying member | 0 (0–3) | 0 (0–3) | 0 (0–3) | 0.802 |
| DOT costs (travel) | 0 (0–0) | 0 (0–0) | 0 (0–0) | 0.506 |
| Indirect costs (household income loss due to TB) | 0 (0–0) | 0 (0–0) | 0 (0–0) | 0.165 |
| Total costs | 8 (5–28) | 8 (5–27) | 8 (5–28) | 0.975 |

IQR = Inter-quartile Range; DOT = Directly Observed Treatment; SLI = Standard of Living Index; USD 1 = INR 70
