# Supplementary material for: Costs incurred by patients with drug-susceptible pulmonary tuberculosis in semi-urban and rural settings of Western India
Source: Infect Dis Poverty. 2020 Oct 19;9:144. doi: 10.1186/s40249-020-00760-w (PMC7574230; doi:10.1186/s40249-020-00760-w)
Supplement: Supplementary file 2 — Additional file 2. Association between type of provider at first visit and catastrophic costs. Table showing statistical association between type of provider at first visit and catastrophic costs incurred by 458 patients with drug-susceptible pulmonary tuberculosis on treatment during January–June 2019. [file 40249_2020_760_MOESM2_ESM.docx]

Supplementary Table 2: Association between type of provider at first visit and catastrophic costs among patients with drug-susceptible pulmonary tuberculosis on treatment during January–June 2019, in Bhavnagar (*n* = 458)

| **Type of provider** | **Catastrophic costs present**  ***n* (%)** | **Catastrophic costs absent**  ***n* (%)** | **Total**  ***n* (%)** |
| --- | --- | --- | --- |
| **Private provider** | 7 (12) | 53 (88) | 60 (100) |
| **Government provider** | 12 (3) | 386 (97) | 398 (100) |
| **Total** | 19 (4) | 439 (96) | 458 (100) |

Pearson chi-squared value = 9.8; Odds ratio (*OR*) = 4.2 [95% *CI*: 1.6–11.3]; *P* = 0.002.
